# Progress in understanding the infection mechanisms, soil microecological imbalance, and integrated control strategies of tobacco black shank

**DOI:** 10.3389/fmicb.2026.1856708

**Published:** 2026-05-28

**Authors:** Xu Wei, Nengfei Tian, Wenqiang Mei, Juan Li, Ming Liu, Haiyang Zhou, Zhijuan Yang, Chengwei Yang, Yanxia Hu

**Affiliations:** 1 Dali Prefecture Branch of Yunnan Tobacco Company, Dali, Yunnan, China; 2 College of Agronomy and Biotechnology, Engineering Research Center of South Upland Agriculture, Ministry of Education, Southwest University, Chongqing, China; 3 Tobacco College, Yunnan Agricultural University, Kunming, Yunnan, China

**Keywords:** biological control, integrated disease management, *phytophthora nicotianae*, precision management, rhizosphere microbiome, soil microecology, soil suppressiveness, tobacco black shank

## Abstract

Tobacco black shank (TBS), caused by the oomycete pathogen *Phytophthora nicotianae*, is a destructive soilborne disease that seriously threatens tobacco production worldwide. This review summarizes recent progress in the infection biology of *P. nicotianae*, the disturbance of rhizosphere microbial communities under disease pressure, and integrated strategies for disease management. Current evidence indicates that TBS development is not only associated with direct pathogen infection, but also with rhizosphere microecological imbalance, including the decline of beneficial microbes, enrichment of opportunistic pathogens, reduced microbial diversity, and weakened soil suppressiveness. These changes may further promote pathogen persistence and disease recurrence. Based on this understanding, effective management should combine crop rotation, biological control, rational chemical intervention, resistant cultivars, and reductive soil disinfestation to suppress pathogen pressure while restoring soil microbial balance. Future research should further integrate multi-omics analysis, microbiome-based regulation, and intelligent monitoring to support early warning and precision control. This review provides an integrated perspective on pathogen–host–soil microbiome interactions and offers a theoretical basis for sustainable management of tobacco black shank.

## 1 Introduction

Tobacco (*Nicotiana tabacum* L.) is an important solanaceous crop with high economic value worldwide, and stable tobacco production is closely associated with agricultural economic development in many tobacco-growing regions (Yang et al., 2023). However, tobacco cultivation is continuously threatened by soilborne diseases throughout the tobacco growth cycle. Among these diseases, tobacco black shank (TBS), caused by the oomycete pathogen *Phytophthora nicotianae*, is widely recognized as one of the most destructive diseases limiting tobacco production (Gai et al., 2023; Cochran et al., 2024). Since its first report in Indonesia in 1896, TBS has spread to most major tobacco-growing areas worldwide (Song et al., 2022). In China, after its introduction in the 1950s, the disease rapidly became an important constraint on tobacco yield, particularly in major production areas such as Yunnan, Guizhou, and Sichuan (Ma et al., 2023).

The severe impact of TBS is closely related to the infection characteristics and ecological adaptability of *P. nicotianae*. Under favorable environmental conditions, the disease can develop rapidly and cause serious root and stem damage, plant wilting, and even plant death. In epidemic years, TBS can lead to yield losses of 30–50%, and complete crop failure may occur in severely affected fields (Zhu et al., 2024). In addition, *P. nicotianae* has a broad host range and can infect plants from more than 90 families and 255 genera, including economically important crops such as tomato and pepper. This broad host range increases its survival potential, dispersal risk, and recurrence in agricultural ecosystems, thereby making long-term disease management more difficult (Cochran et al., 2024).

In addition to causing direct plant damage, TBS is increasingly recognized as a soil microecology-related disease. Previous studies have shown that invasion by *P. nicotianae* is often accompanied by marked disturbances in rhizosphere microbial communities, which may further weaken the natural disease-suppressive capacity of soils and promote disease recurrence (Ma J. et al., 2024). Therefore, the occurrence of TBS is not only determined by the interaction between host plants and pathogens, but is also closely associated with soil environmental conditions and rhizosphere microbial community dynamics. This understanding provides an important basis for shifting disease management from simple pathogen suppression toward soil microecological regulation.

Traditional management of tobacco black shank has long relied on chemical fungicides such as metalaxyl. Although chemical fungicides can provide rapid disease suppression, long-term and large-scale application of single fungicides may accelerate the development of pathogen resistance and disturb soil microbial community structure, thereby reducing soil ecological stability and disease-suppressive functions (Ma et al., 2023; Tang et al., 2023). With the transition of modern agriculture toward greener and more sustainable practices, TBS management is gradually shifting from pathogen eradication alone to integrated microecological regulation. This strategy emphasizes the reconstruction of a healthy soil microbiome and the enhancement of plant-associated defense capacity through coordinated agronomic, biological, chemical, and technological approaches (Ma X. et al., 2024; Lu et al., 2025).

**FIGURE 1:**
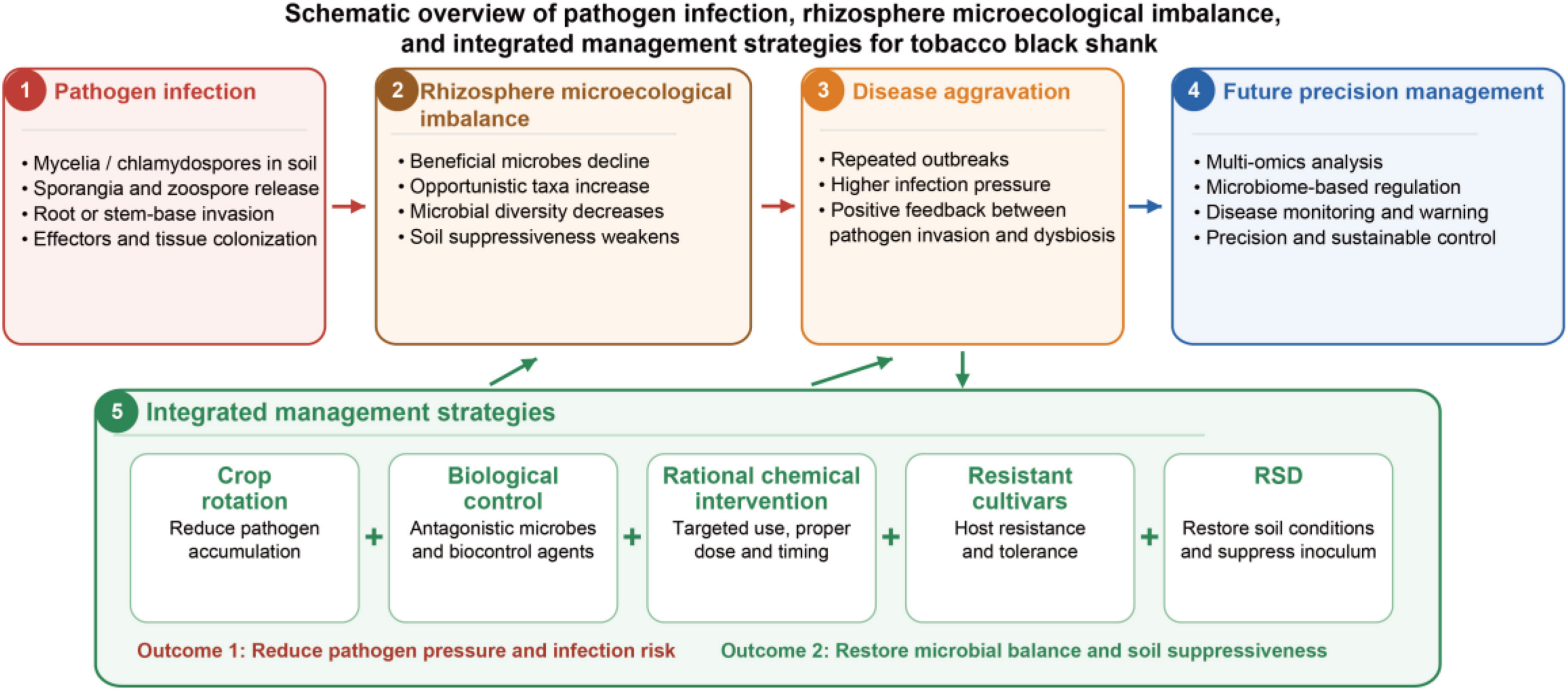
Schematic overview of pathogen infection, rhizosphere microecological imbalance, and integrated management strategies for tobacco black shank. The figure summarizes the main framework of this review. *Phytophthora nicotianae* survives in soil through persistent propagules and initiates infection through zoospore-mediated host invasion. Pathogen infection not only damages host tissues directly, but also disturbs rhizosphere microbial communities, leading to the depletion of beneficial microorganisms, enrichment of opportunistic pathogens, reduced microbial diversity, and weakened soil suppressiveness. These changes can further aggravate disease development. To interrupt this process, integrated management strategies, including crop rotation, biological control, rational chemical intervention, resistant cultivars, and reductive soil disinfestation, are required. Future management should further integrate multi-omics analysis, microbiome-based regulation, and intelligent monitoring to improve the precision and sustainability of tobacco black shank control.

Despite increasing progress in understanding the pathogenicity of *P. nicotianae* and developing control measures for TBS, current knowledge remains relatively fragmented. Unlike previous reviews that mainly summarized pathogen biology or individual control measures, this review places greater emphasis on the links among pathogen infection, rhizosphere microbiome disruption, loss of soil suppressiveness, and system-level disease management. It also highlights how multi-omics tools and intelligent monitoring may be incorporated into practical decision-making for tobacco black shank control. Therefore, this review synthesizes current knowledge on the biological characteristics and infection mechanisms of *P. nicotianae*, the responses of rhizosphere and soil microbial communities under disease pressure, and the major strategies used for disease management, including crop rotation-mediated regulation, biological control, chemical-assisted approaches, and intelligent diagnostic technologies. By integrating recent advances in metagenomics, proteomics, and artificial intelligence, this review aims to establish an integrated perspective on pathogen–host–soil microbiome interactions and provide a theoretical basis for precise and sustainable management of tobacco black shank. The overall framework of this review is summarized in Figure 1.

## 2 Pathogen infection mechanisms and soil microbial community dynamics in tobacco black shank

The biological characteristics and infection mechanisms of the tobacco black shank pathogen are highly specialized, and a thorough understanding of these features is essential for developing effective disease management strategies. *Phytophthora nicotianae* exhibits strong survival capacity and ecological adaptability, displaying multiple morphological forms throughout its life cycle (Yuan et al., 2021; Cochran et al., 2024). In soil environments, the pathogen primarily overwinters as dormant mycelia and chlamydospores, which serve as the primary inoculum for disease outbreaks in the following growing season (Yuan et al., 2021; Zhu et al., 2024). Under favorable temperature and moisture conditions, chlamydospores germinate to produce germ tubes that subsequently develop into mycelia or form sporangia, which release motile zoospores.

In addition to its survival structures and infection cycle, population variation in *P. nicotianae* is also important for explaining regional differences in disease severity and control efficacy. Field populations of *P. nicotianae* may differ in virulence, host adaptation, environmental fitness, and fungicide sensitivity (Zhang et al., 2003; Li et al., 2017). Such variation can influence disease severity, the durability of resistant cultivars, and the effectiveness of chemical control. Pathotype differentiation is particularly relevant because cultivars that perform well against ase severity, the durability of resistant cultivars, and the effectiveness of chemical control. Pathotype differentiation is particularly relevant because cultivars that perform well against one pathogen population may show reduced resistance when exposed to more aggressive or genetically distinct isolates (Gallup et al., 2018). The reported occurrence of metalaxyl-resistant *P. nicotianae* also indicates that pathogen populations can adapt under long-term fungicide selection pressure (Miller et al., 2024). Therefore, monitoring the genetic diversity, virulence variation, and fungicide sensitivity of *P. nicotianae* populations should be considered an important component of regional disease management. However, compared with studies on disease symptoms and control measures, systematic information on the population genetics and pathotype structure of tobacco-associated *P. nicotianae* remains limited and requires further investigation.

The infection process begins when zoospores move through water films in the soil toward tobacco roots or stem bases. They penetrate host tissues either directly through epidermal cells or via natural openings and small wounds (Van West et al., 2002). Following successful colonization, the pathogen secretes a variety of cell wall-degrading enzymes and effector proteins to suppress plant defense responses, thereby facilitating infection (Yuan et al., 2021). As hyphae expand within cortical tissues and the vascular system, the transport of water and nutrients is impaired, resulting in progressive wilting and tissue collapse. The pathogen-induced damage is further associated with browning and shrinkage of the stem pith, which often produces the characteristic “disc-like” appearance. As disease development progresses, water-soaked dark brown lesions appear at the stem base and extend upward, eventually causing blackened and decayed stem tissues, from which the disease name “black shank” is derived (Wheller et al., 1998). Disease development is strongly favored by warm and wet soil conditions because soil water is required for sporangium formation, zoospore release, and zoospore movement toward host tissues. Poorly drained fields, prolonged rainfall, excessive irrigation, and high soil moisture can increase the duration of root and stem-base exposure to infective propagules, thereby accelerating disease spread (Pandey, 2023; Cochran et al., 2024). Temperature also affects epidemic development by regulating mycelial growth, sporulation, zoospore activity, and infection rate. Under suitable warm conditions, the pathogen can colonize host tissues more rapidly, whereas unfavorable temperatures may slow pathogen growth and delay symptom development. Continuous cropping further increases epidemic risk by promoting the accumulation of soilborne inoculum and reducing opportunities to interrupt the pathogen life cycle. Therefore, field outbreaks are most likely when high inoculum density, susceptible cultivars, warm soil conditions, and prolonged soil moisture occur simultaneously. Conversely, improved drainage, reduced periods of soil saturation, and appropriate irrigation management can limit zoospore movement and reduce infection opportunities.

In recent years, increasing evidence has revealed that pathogen invasion not only directly damages host plants but also profoundly reshapes the structure, function, and ecological networks of rhizosphere and bulk soil microbial communities, thereby forming a self-reinforcing vicious cycle (Cao et al., 2026). One of the most notable impacts of pathogen invasion is the widespread decline in soil microbial α-diversity. Studies based on 16S rRNA gene and ITS sequencing have shown that both bacterial and fungal diversity indices are significantly lower in soils with high incidence of tobacco black shank compared with healthy soils (Shen et al., 2018). Importantly, this loss of diversity is not random but highly selective (Mendes et al., 2018). Beneficial bacterial taxa with biocontrol potential, including *Pseudomonas, Bacillus*, and *Streptomyces*, show a sharp decline in relative abundance in infected soils (Luo et al., 2019; Liang J. et al., 2024). These beneficial microorganisms often inhibit pathogens by producing antibiotics, siderophores, and other antagonistic compounds; thus, their reduction may weaken the natural disease-suppressive capacity of soils (Haas and Défago, 2005).

At the same time, certain opportunistic pathogens or potentially pathogenic microorganisms, such as species of *Fusarium*, tend to become enriched, further aggravating the deterioration of the rhizosphere microenvironment (Trivedi et al., 2017; Liang J. et al., 2024). Analyses of microbial community composition following pathogen invasion also reveal notable shifts in fungal community structure. Specifically, the relative abundance of *Ascomycota* often increases, whereas *Basidiomycota* decreases in diseased soils (Xiong et al., 2017; Goncharov et al., 2020; Zhang et al., 2021). These findings suggest that infection by *P. nicotianae* is not an isolated event but rather triggers cascading responses within the soil microecosystem, including the decline of beneficial microbes, proliferation of pathogens, reduced community diversity, and fragmentation of ecological networks (Liu et al., 2024; Zhang et al., 2024).

The depletion of beneficial microbial taxa, together with the enrichment of opportunistic or pathogenic microorganisms, may impair microbial interactions and reduce the functional stability of disease-suppressive soils (Ding et al., 2021; Jiang et al., 2025). Such changes may create ecological conditions that favor the persistence and proliferation of *P. nicotianae*, thereby increasing the risk of repeated disease outbreaks. In turn, disease development further reshapes the rhizosphere microbial community, leading to a feedback process in which pathogen invasion, microbial dysbiosis, and disease aggravation reinforce one another. This ecological perspective indicates that tobacco black shank management should move beyond pathogen-centered suppression and incorporate soil microecological restoration. Accordingly, integrated approaches that combine crop rotation, biological control, and rational chemical intervention are needed to reduce inoculum pressure, rebuild beneficial microbial communities, and enhance the intrinsic suppressive capacity of soil.

It should be noted that many current studies on TBS-associated microbiomes are based on field surveys or sequencing-based correlation analyses. Therefore, changes in microbial diversity or community composition may represent both drivers and consequences of disease development. Although the depletion of beneficial taxa and enrichment of opportunistic microorganisms are frequently associated with diseased soils, direct causal relationships require further validation through microbial isolation, synthetic community reconstruction, pathogen challenge assays, and field re-inoculation experiments. This distinction is important for translating microbiome observations into reliable disease management strategies.

## 3 Integrated management strategies for tobacco black shank

The sustainable management of tobacco black shank requires strategies that can suppress *Phytophthora nicotianae* while maintaining soil ecological stability. Because this disease is closely associated with pathogen survival, host susceptibility, soil environmental conditions, and rhizosphere microbial community structure, reliance on a single control measure is often insufficient. Current management strategies mainly include crop rotation, biological control, rational chemical control, resistant cultivars, and soil microecological restoration. These approaches differ in their mechanisms and application scenarios, but their coordinated use provides a more practical pathway for both short-term disease suppression and long-term improvement of soil suppressiveness.

### 3.1 Crop rotation and regulation of soil microbial communities

Crop rotation is an important agronomic strategy for controlling soilborne diseases because it can interrupt host continuity, alter rhizosphere nutrient conditions, and reshape soil microbial communities (Wang et al., 2015; Jin et al., 2019). In tobacco black shank management, rotation reduces the continuous supply of suitable host tissues for *P. nicotianae*, thereby limiting pathogen accumulation in soil. At the same time, different rotation crops release distinct root exudates, which can modify the rhizosphere microenvironment and influence pathogen survival, zoospore behavior, and microbial community assembly.

Several rotation systems have been reported to reduce tobacco black shank incidence compared with continuous tobacco cropping. Rotations involving rapeseed, wheat, rice, soybean, or other non-host or antagonistic crops can suppress disease development by reducing pathogen pressure and changing soil microbial composition. For example, rapeseed roots can attract zoospores and release antimicrobial compounds, thereby interfering with pathogen infection and reducing disease incidence in the field (Fang et al., 2016). These findings suggest that crop rotation is not only a physical interruption of the pathogen life cycle, but also a biological regulation process mediated by root exudates and rhizosphere interactions.

Crop rotation also contributes to the reconstruction of disease-suppressive microbial communities. Compared with continuous tobacco cropping, tobacco–marigold rotation has been shown to increase bacterial and fungal diversity and promote the enrichment of beneficial bacterial groups in the soil microbial community (Huang et al., 2022). Such changes may enhance microbial competition against pathogens, improve ecological network stability, and strengthen the natural suppressive capacity of soil. In addition, crop rotation can improve soil structure and fertility by increasing soil organic matter and nutrient availability, which indirectly supports the development of a more stable rhizosphere microenvironment (Liu et al., 2025).

However, the effectiveness of crop rotation is strongly dependent on crop species, rotation duration, local climate, soil type, and initial pathogen density. Short rotation periods or unsuitable rotation crops may provide limited suppression and, in some cases, may fail to reduce disease pressure effectively (Gai et al., 2023). Moreover, crop rotation cannot completely eliminate dormant survival structures of *P. nicotianae*, especially long-lived chlamydospores in soil (Pandey, 2023). Therefore, crop rotation should be regarded as a foundational measure for reducing long-term disease risk rather than a stand-alone solution for heavily infested fields. In practice, it should be combined with resistant cultivars, biological control, rational chemical treatment, or soil disinfestation to achieve more stable disease management effects (Yan et al., 2023).

### 3.2 Application of biological control strategies

Biological control has received increasing attention as an environmentally compatible strategy for tobacco black shank management. Its main principle is to suppress *P. nicotianae* through antagonism, competition, induced host resistance, and regulation of the rhizosphere microbial community. Compared with chemical fungicides, biological control agents generally have lower environmental risks and are more consistent with sustainable tobacco production systems (Ma et al., 2023).

Among bacterial biocontrol agents, *Bacillus* species are widely studied because of their strong environmental adaptability, spore-forming ability, and capacity to produce antimicrobial metabolites. They can inhibit pathogen growth, compete for nutrients and ecological niches, promote plant growth, and induce systemic resistance in host plants (Serrão et al., 2024). For example, *Bacillus velezensis* Ba168, isolated from tobacco field soil, showed strong antagonistic activity against *P. nicotianae* and demonstrated promising control effects in both laboratory and field experiments (Guo et al., 2020).

Fungal biocontrol agents also play important roles in tobacco black shank control. *Trichoderma* species are among the most commonly studied fungi because they can inhibit pathogens through mycoparasitism, competition, production of antifungal compounds, and stimulation of plant defense responses. *Trichoderma reesei* ZP2-1, isolated from tobacco rhizosphere soil, has been reported to exhibit significant antagonistic activity against *P. nicotianae* (Peng et al., 2018). In addition, actinomycetes are important sources of antibiotics and other bioactive metabolites. For instance, *Streptomyces albidoflavus* S20 showed a 90.3% inhibition rate against *P. nicotianae* in plate confrontation assays, and pot and field experiments achieved control efficiencies of 92.1 and 90.4%, respectively (Wang et al., 2025).

Although single strains can show strong antagonistic effects under controlled conditions, their performance in the field is often unstable. This instability is mainly associated with poor rhizosphere colonization, competition with indigenous microorganisms, sensitivity to temperature and moisture, and possible incompatibility with pesticides. To improve field performance, microbial consortia and mixed microbial formulations have been proposed. Compared with single strains, microbial consortia may provide broader functional coverage and greater ecological stability under complex field conditions (Liang F. et al., 2024). For example, the combined application of chitosan oligosaccharides and *Bacillus amyloliquefaciens* improved the control efficacy against tobacco black shank through multiple mechanisms, including pathogen inhibition and enhancement of plant defense responses (Zeng et al., 2023).

Therefore, the future application of biological control should focus not only on screening highly antagonistic strains, but also on improving colonization ability, formulation stability, carrier materials, application timing, and compatibility with agronomic and chemical measures. Biological control is most effective when it is incorporated into an integrated management system rather than used as an isolated replacement for other control strategies.

### 3.3 Advantages, limitations, and rational use of chemical control

Chemical control remains an important component of tobacco black shank management because of its rapid and relatively reliable disease suppression, especially when disease pressure is high or early symptoms have already appeared (Duan et al., 2025). Fungicides can directly inhibit pathogen growth by targeting key physiological processes such as cell wall biosynthesis, membrane integrity, respiration, or energy metabolism. Therefore, chemical control is still valuable for emergency intervention and for preventing rapid disease spread during epidemic periods.

However, the use of chemical fungicides also presents clear ecological and practical risks. Long-term or excessive application may disturb non-target soil microbial communities, reduce beneficial microbial populations, and weaken soil ecological functions. More importantly, repeated use of fungicides with the same mode of action can accelerate the development of resistant *P. nicotianae* populations. This concern has long been recognized in tobacco black shank control, particularly for fungicides such as metalaxyl (Li et al., 2008; Wang et al., 2011). Resistance to metalaxyl has already been reported in several tobacco-growing regions, indicating that chemical control alone is not sustainable (Zhu et al., 2007; Xu et al., 2008).

Recent studies have therefore emphasized more environmentally compatible chemical-assisted strategies. Plant-derived compounds are of particular interest because of their relatively low toxicity and potential compatibility with ecological disease management. For example, magnolol from *Magnolia officinalis* can inhibit *P. nicotianae* by disrupting redox homeostasis and energy metabolism (Wang X. et al., 2022). Quercetin, a natural flavonoid compound, has been shown to enhance tobacco resistance to *P. nicotianae* by activating defense responses, including antioxidant enzyme activity and secondary metabolite accumulation (Duan et al., 2025). These compounds may not fully replace conventional fungicides in all field situations, but they provide useful options for reducing chemical pressure and improving host resistance.

In addition to plant-derived compounds, micronutrient-based regulation has also shown potential in tobacco black shank management. Molybdenum is involved in plant growth, photosynthesis, nitrogen metabolism, and antioxidant processes, while selenium can improve plant stress resistance and defense-related metabolism. Field studies have shown that soil application of 0.6 mg/kg molybdenum significantly reduced the incidence of tobacco black shank (Yu et al., 2024), and foliar spraying with 8 mg/L selenium also reduced disease occurrence (Ma X. et al., 2024). These findings indicate that chemical-assisted control can be broadened from direct pathogen inhibition to host resistance enhancement and physiological regulation.

Nevertheless, conventional fungicides such as metalaxyl and dimethomorph remain widely used in field production. Their use should be based on disease monitoring, pathogen sensitivity, field risk assessment, and rotation of fungicide modes of action. Studies have shown that metalaxyl–mancozeb can significantly reduce soil bacterial and fungal diversity and decrease beneficial microbial groups such as *Bacillus* and *Pseudomonas* (Wang Y. et al., 2022). Therefore, chemical control should be applied precisely and rationally, with the goal of suppressing disease outbreaks while minimizing negative effects on soil microbial communities. Balancing rapid disease suppression with ecological safety remains a key challenge for integrated disease management.

### 3.4 Integrated disease management

Because tobacco black shank is driven by pathogen infection, host susceptibility, environmental conditions, and soil microbial imbalance, no single control measure can simultaneously provide rapid disease suppression and long-term soil health improvement. Integrated disease management should therefore combine resistant cultivars, crop rotation, biological control, chemical intervention, and soil microecological restoration according to field disease pressure and soil conditions.

Resistant cultivars are a basic component of integrated management because they reduce the probability of successful pathogen infection at the host level. Resistance to *P. nicotianae* is associated with gene expression regulation, defense-related metabolic changes, and physiological responses in tobacco plants (Wang W. et al., 2022; Wen et al., 2023). However, the effectiveness of resistant cultivars is not absolute. Their resistance may vary with pathogen population structure, pathotype composition, environmental conditions, and field inoculum density. In areas with high disease pressure or favorable conditions for epidemic development, resistant cultivars may still become infected. In addition, the long-term deployment of cultivars with similar resistance backgrounds may impose selection pressure on *P. nicotianae* populations and reduce resistance durability. Therefore, resistant cultivars should be used as a key component of integrated management rather than as a single control measure.

At the preventive and early infection stages, biological control agents such as *Bacillus* and *Pseudomonas* can be applied to promote rhizosphere colonization before extensive pathogen infection occurs (Ma et al., 2023). These beneficial microorganisms can suppress *P. nicotianae* through nutrient and niche competition, production of antimicrobial compounds, and induction of plant systemic resistance (Zeng et al., 2023; Zhang et al., 2026). When disease symptoms begin to appear or when field disease risk is high, precise chemical intervention may be necessary. Fungicides such as fluopicolide and metalaxyl can rapidly suppress pathogen activity and help prevent disease spread (Webb and Bailey, 2024). Plant-derived compounds such as magnolol and quercetin may also contribute to pathogen suppression or host resistance enhancement (Wang X. et al., 2022; Duan et al., 2025). These chemical measures should be used as targeted interventions rather than routine applications, thereby reducing selection pressure for fungicide resistance and limiting disturbance to soil microbial communities (Ahumada et al., 2025).

For long-term disease management, the key objective is to reduce soilborne inoculum and rebuild disease-suppressive soil microecosystems. Crop rotation plays an important role in this process by interrupting host continuity, reducing pathogen accumulation, and altering root exudate-mediated microbial recruitment (Zhang et al., 2020; Huang et al., 2022). For example, tobacco–marigold and tobacco–rapeseed rotation systems can reduce pathogen pressure and promote beneficial microbial taxa in the rhizosphere (Huang et al., 2022; Gai et al., 2023). These effects provide a biological basis for restoring soil suppressiveness in continuous tobacco cropping systems.

Reductive soil disinfestation (RSD) is another promising approach for heavily infested soils. By incorporating organic materials and creating anaerobic conditions, RSD can suppress aerobic soilborne pathogens while stimulating the activity of certain indigenous microorganisms (Qin et al., 2023; Qingshan et al., 2025). The effectiveness of RSD is strongly affected by the type and carbon-to-nitrogen ratio of organic amendments. High C/N substrates may sustain microbial activity for longer periods and prolong pathogen suppression (Luo et al., 2024). After soil conditions are improved through rotation or RSD, the introduction of well-characterized biocontrol strains may further reinforce the rhizosphere microbial barrier.

**FIGURE 2:**
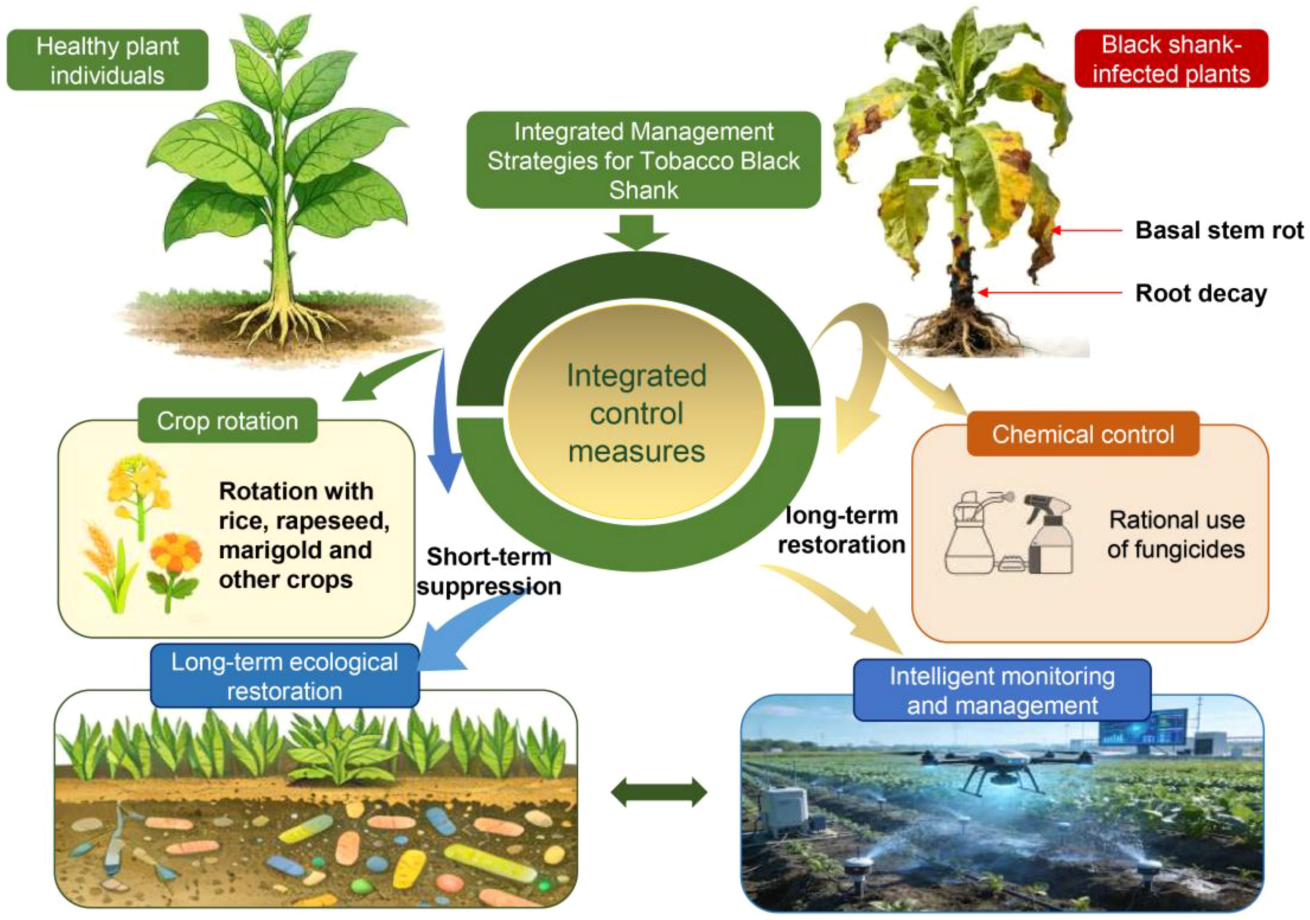
Conceptual framework for integrated management of tobacco black shank. The framework illustrates the coordinated control of tobacco black shank through pathogen suppression, host resistance enhancement, soil microecological restoration, and precision management. Resistant cultivars and biological control agents reduce the initial risk of *Phytophthora nicotianae* infection, while rational chemical intervention provides rapid disease suppression during high-risk periods. Crop rotation and reductive soil disinfestation contribute to the reduction of soilborne inoculum and the reconstruction of disease-suppressive microbial communities. Multi-omics analysis and intelligent monitoring further support disease risk assessment and precision decision-making, thereby linking short-term disease control with long-term soil health improvement.

Overall, integrated management of tobacco black shank should be understood as a staged and coordinated strategy. Resistant cultivars and biological control help reduce initial infection risk; precise chemical intervention provides rapid suppression during high-risk periods; crop rotation and RSD reduce pathogen reservoirs and improve the soil environment; and microbial regulation strengthens soil suppressiveness over the long term. This integrated framework links short-term disease control with long-term soil health restoration and provides a more sustainable approach for managing tobacco black shank. To make integrated management more applicable to field production, disease control measures can be organized according to different epidemic stages, as summarized in Table 1. The conceptual framework of integrated management strategies for tobacco black shank is shown in Figure 2.

**TABLE 1:** Stage-based practical framework for integrated management of tobacco black shank.

Disease stage	Main objective	Recommended actions
Preventive stage	Reduce inoculum and improve soil suppressiveness	Crop rotation; resistant cultivars; soil drainage improvement; RSD in heavily infested fields; organic amendments; biocontrol agent application before transplanting
Early infection stage	Suppress initial infection and prevent spread	Apply biocontrol agents; targeted fungicide application; improve drainage; remove severely infected plants; monitor symptoms
Severe outbreak stage	Limit yield loss and reduce carryover inoculum	Emergency chemical control; field sanitation; drainage management; avoid moving contaminated soil; post-harvest residue removal; plan rotation or RSD for the next season

## 4 Challenges and future perspectives

### 4.1 Current research bottlenecks

Although substantial progress has been made in the management of tobacco black shank, several key challenges still limit the development of stable and sustainable control strategies. One major bottleneck is the inconsistent field performance of biological control agents. Many strains with strong antagonistic activity against *Phytophthora nicotianae* have been identified under laboratory or greenhouse conditions, but their control efficacy often declines or fluctuates in open-field environments. This instability is closely related to the complexity of soil microbial communities, environmental heterogeneity, and the limited ecological adaptability of introduced microorganisms. Panchal et al. (2026) suggested that the instability of traditional single-strain inoculants is largely associated with complex microbial interactions, weak adaptation to local soil conditions, competitive disadvantages against indigenous microbial communities, and narrow functional capacity.

The limitations of single-strain inoculants also reflect the ecological complexity of disease-suppressive microbiomes. Many beneficial functions in the rhizosphere are not performed by isolated microorganisms alone, but depend on cooperative interactions among multiple microbial taxa. In complex soil systems, single strains may have difficulty establishing stable populations and maintaining consistent functional expression (Xiong, 2009). Recent work by Song et al. (2026) further demonstrated functional division within synthetic microbial communities, indicating that coordinated microbial interactions may provide greater stability than single-strain applications. These findings suggest that future biological control should move from simple strain screening toward the construction and validation of functionally complementary microbial consortia.

Another important challenge is the incomplete understanding of pesticide impacts on soil microbial communities and ecological functions. Although chemical fungicides remain important for rapid disease suppression, their non-target effects vary substantially across soil types, environmental conditions, and management systems. Némethová et al. (2026) reported that pesticide effects on soil bacterial communities are strongly environment-dependent, with soil pH explaining more than 60% of microbial community variation and exerting a stronger influence than pesticide type itself. This finding indicates that pesticide risk assessment should not focus only on active ingredients, but should also consider soil physicochemical background and microbial community context.

Similarly, Li et al. (2026) showed that pesticide application significantly altered microbial community structure and nitrogen transformation processes in tobacco rhizosphere soil, and that different pesticides had distinct effects on xenobiotic degradation pathways. These results highlight the need to evaluate chemical control not only by its direct disease-suppressive efficacy, but also by its effects on soil nutrient cycling, microbial functional stability, and long-term soil health.

A further methodological bottleneck lies in identifying functional microorganisms and causal regulatory targets from large-scale microbiome datasets. Current studies often describe correlations between microbial taxa and disease incidence, but correlation alone is insufficient to determine whether specific microorganisms directly contribute to disease suppression. Therefore, future research needs stronger causal validation frameworks, including synthetic community construction, microbial isolation and re-inoculation, functional gene verification, and field-scale validation. Establishing these links is essential for translating microbiome data into practical disease management strategies.

### 4.2 Future research directions

Future research on tobacco black shank should shift from single-factor control toward integrated regulation of the pathogen–host–soil microbiome system. Conventional biological control mainly relies on the introduction of exogenous beneficial microorganisms. However, introduced strains often face poor colonization, limited persistence, and strong competition from native microbial communities. Therefore, an important direction is to develop strategies that not only introduce beneficial agents, but also create soil conditions that support their establishment and function.

One promising direction is the use of prebiotic-based soil microecological regulation. Unlike traditional inoculation strategies, prebiotic approaches aim to activate indigenous beneficial microorganisms by supplying specific substrates or modifying the rhizosphere environment. This strategy may guide soil microbial communities toward a more disease-suppressive state while reducing dependence on exogenous strains. For example, agrobiomass-derived xylooligosaccharides have been shown to selectively promote beneficial microbial growth and suppress pathogen proliferation, suggesting their potential as soil microecological regulators (Chadathong et al., 2025). Compared with direct microbial inoculation, this approach may be more compatible with native soil ecosystems and may provide more stable disease control effects.

Another important direction is the integration of multi-omics technologies with field-based disease monitoring. Metagenomics, metatranscriptomics, metabolomics, and proteomics can provide complementary information on microbial composition, functional potential, active metabolic processes, and host defense responses. When combined with field disease data, these approaches can help identify key microbial taxa, functional genes, metabolites, and host pathways associated with disease resistance or susceptibility. Such information is critical for moving from descriptive microbiome studies toward mechanism-based disease management. Soil metagenomics is also increasingly moving beyond community description toward predictive assessment of soil health and plant–microbe interactions. By linking microbial community features with field disease performance, predictive models may help identify high-risk fields before visible symptoms appear and support earlier intervention (Choi et al., 2026).

For tobacco black shank, AI-based monitoring and predictive systems should be developed around real field disease scenarios rather than being treated only as general crop disease-recognition tools. In the short term, image-based models may assist in identifying typical aboveground symptoms, such as plant wilting and field-level disease patches, as well as visible stem-base lesions when infected tissues are exposed. Recent models such as SPROUT and SIS-YOLOv8 demonstrate the potential of few-shot learning and improved object detection for crop disease diagnosis under limited samples or complex field conditions (Venkatraman et al., 2026; Qin et al., 2025). However, their direct application to tobacco black shank still requires disease-specific image datasets, standardized symptom annotation, and validation under different tobacco-growing environments.

In practical field management, AI-assisted risk prediction should integrate multiple data sources, including rainfall, temperature, soil moisture, drainage conditions, soil physicochemical properties, cropping history, cultivar resistance, and pesticide application records. These data can be used to construct regional disease-risk models and support decisions after prolonged rainfall, excessive irrigation, or other high-risk conditions. For example, such models could help determine whether drainage improvement, biocontrol application, or targeted fungicide treatment is needed in specific fields. In the medium term, pathogen detection data, such as qPCR-based quantification of *P. nicotianae*, should be incorporated into these models to estimate soilborne inoculum density before transplanting or during early disease development.

In the longer term, multi-omics datasets may help identify microbiome or metabolite indicators associated with disease-suppressive and disease-conducive soils. These indicators could be used for soil health assessment and microbiome-based regulation. When combined with machine learning algorithms and field management records, they may support site-specific recommendations, including optimized rotation systems, compatible biocontrol combinations, and threshold-based pesticide application (Portal-Gonzalez et al., 2025). However, practical implementation still faces several barriers, including the limited availability of well-annotated tobacco black shank image datasets, difficulty in detecting belowground early infection, inconsistent field records among regions, high costs of pathogen and microbiome monitoring, and limited model transferability across different ecological zones.

Therefore, AI-assisted management of tobacco black shank should be developed stepwise. The short-term priority is to establish standardized field datasets, including disease images, meteorological records, soil moisture, drainage status, cropping history, and management information. The medium-term goal is to build regional disease-risk models by integrating field monitoring with pathogen detection. The long-term goal is to combine multi-omics indicators, microbiome-based regulation, and intelligent decision-support systems to improve early warning, precision intervention, and sustainable disease management. Overall, future control of tobacco black shank should not only aim to suppress *P. nicotianae*, but also to maintain microbial functional stability, reduce unnecessary chemical disturbance, and enhance soil suppressiveness.

## 5 Conclusion

Tobacco black shank is not only a disease caused by *Phytophthora nicotianae* infection, but also a soilborne disease closely associated with rhizosphere microecological imbalance. The pathogen damages host tissues directly and alters rhizosphere microbial community structure, leading to the decline of beneficial microorganisms, enrichment of opportunistic pathogens, and weakening of soil suppressiveness. These changes can further promote pathogen persistence and disease recurrence, highlighting the need to understand tobacco black shank within the framework of pathogen–host–soil microbiome interactions.

Current management strategies, including crop rotation, biological control, rational chemical intervention, resistant cultivars, and reductive soil disinfestation, each contribute to disease suppression through different mechanisms. Crop rotation and RSD help reduce pathogen reservoirs and restore soil microbial balance; biological control enhances antagonistic and plant defense-related functions; chemical control provides rapid intervention during high-risk periods; and resistant cultivars reduce host susceptibility. However, no single measure can simultaneously ensure immediate disease control and long-term soil health improvement.

Therefore, sustainable management of tobacco black shank should rely on an integrated strategy that combines pathogen suppression with soil microecological restoration. Future control systems should emphasize resistant cultivars, optimized rotation systems, stable biocontrol agents, precise chemical use, and microbiome-based monitoring. This integrated perspective provides a theoretical basis and practical direction for improving the precision and sustainability of tobacco black shank management.
